# Conservation of Prion-Like Composition and Sequence in Prion-Formers and Prion-Like Proteins of *Saccharomyces cerevisiae*

**DOI:** 10.3389/fmolb.2019.00054

**Published:** 2019-07-11

**Authors:** Ting-Yi Su, Paul M. Harrison

**Affiliations:** Department of Biology, McGill University, Montreal, QC, Canada

**Keywords:** prion, evolution, sequence analysis, fungi, disease, glutamine, asparagine

## Abstract

Prions in eukaryotes have been linked to diseases, evolutionary capacitance, large-scale genetic control, and long-term memory formation. Prion formation and propagation have been studied extensively in the budding yeast *Saccharomyces cerevisiae*. Here, we have analysed the conservation of sequence and of prion-like composition for prion-forming proteins and for other prion-like proteins from *S. cerevisiae*, across three evolutionary levels. We discover that prion-like status is well-conserved for about half the set of prion-formers at the *Saccharomycetes* level, and that prion-forming domains evolve more quickly as sequences than other prion-like domains do. Such increased mutation rates may be linked to the acquisition of functional roles for prion-forming domains during the evolutionary epoch of *Saccharomycetes*. Domain scores for prion-like composition in *S. cerevisiae* are strongly correlated with scores for such composition weighted evolutionarily over the dozens of fungal species examined, indicating conservation of such prion-like status. Examples of notable prion-like proteins that are highly conserved both in sequence and prion-like composition are discussed.

## Introduction

Prions are proteinaceous infectious particles that were originally identified as the causative agents (made from the prion protein PrP) of devastating neurological diseases in mammals. Prions propagate alternative protein states, through co-option of further copies of the same proteins. In budding yeast (*Saccharomyces cerevisiae*), propagation of these alternative states can be sustained during budding, mating, and artificial laboratory protocols. Such yeast prions have been linked to diverse phenomena including evolutionary capacitance, disease-like states, and large-scale genetic control. The first well-characterized yeast prions, that underlie the [PSI+] and [URE3] prions, are propagating amyloids of the proteins Sup35p and Ure2p, respectively. The protein Sup35p is part of the translation termination complex. [PSI+] prion formation reduces translation termination efficiency and increases non-sense-codon read-through levels (Cox, 1965; Shorter and Lindquist, 2005). This read-through has been shown to have a potential role in uncovering cryptic genetic variation (True and Lindquist, 2000; True et al., 2004). [URE3] causes upregulation of poor nitrogen source usage, even when rich sources are available (Lacroute, 1971; Wickner, 1994; Wickner et al., 2004). Prion variants sometimes behave as budding-yeast diseases (Nakayashiki et al., 2005; McGlinchey et al., 2011). The [MOT3+] prion has been shown to have a possible role in control of transitions to multicellularity (Holmes et al., 2013). The stress-inducible cytoskeleton-linked budding-yeast protein Lsb2 can form a metastable prion in response to high temperatures (Chernova et al., 2017). There are now >10 known amyloid-based prions of *S. cerevisiae* (Harbi et al., 2012; Harbi and Harrison, 2014). Prion-forming proteins have also been discovered in the fungus *Podospora anserina* and the fission yeast *Schizosaccharomyces pombe* (Saupe, 2011; Sideri et al., 2017). Almost all amyloid-based budding yeast prion-forming regions have a high degree of intrinsic disorder and share a bias for asparagine (N) and/or glutamine (Q) residues (Harbi and Harrison, 2014; Harrison, 2017). Glutamine and asparagine have differing influences on prion formation: Ns promote benign prion formation, whereas excess Q can lead to toxic non-amyloid conformer formation (Halfmann et al., 2011). Several algorithms have been developed that annotate protein regions with high potential prion-forming propensity (Espinosa Angarica et al., 2013; Ross et al., 2013; Lancaster et al., 2014; Zambrano et al., 2015). Prion-like proteins in yeast and other organisms have more recently been linked to other processes, such as the formation of stress granules and other membraneless biomolecular condensates (Jain et al., 2016; Franzmann et al., 2018).

The original mammalian PrP domain is not biased for Ns and Qs, and is deeply conserved since a PrP founder gene emerged in early chordate evolution (Harrison et al., 2010; Ehsani et al., 2011; Westaway et al., 2011). The [PSI+] prion has an N/Q bias that is conserved across *Ascomycota* and *Basidiomycota*, which diverged >1 billion years ago (Harrison et al., 2007). A large population of yeast-prion-like proteins emerged early in the evolution of *Saccharomycetes*, as a result of mutational trends to form more polyasparagine runs, thus providing an evolutionary “test set” from which several prion-forming domains seem to have developed (An and Harrison, 2016). Eukaryotic proteomes often bear large numbers of these prion-like domains. The slime mold *Dictyostelium* has >20% of its proteins containing prion-like domains (Malinovska et al., 2015; An et al., 2016) and there is evidence it has evolved a mechanism for subvertion of prion formation (Malinovska and Alberti, 2015; Malinovska et al., 2015). Other organisms that have high levels of prion-like proteins include *Drosophila melanogaster, Plasmodium falciparum*, and the leech *Helobdella robusta* (An et al., 2016; Pallarès et al., 2018). Several other yeast-prion-like proteins have links to human neurodegenerative pathomechanisms (Sun et al., 2011; Kim et al., 2013; Pokrishevsky et al., 2016) or to long-term memory formation in *Aplysia* and *Drosophila* (Si et al., 2010; Khan et al., 2015). Predicted prions have been detected in all the domains of life (Espinosa Angarica et al., 2013), including thousands in viruses and phages (Tetz and Tetz, 2017, 2018), and tens of thousands in bacteria (Harrison, 2019). Possible bacterial prion-forming proteins have also been detected experimentally (Yuan et al., 2014; Shahnawaz et al., 2017; Yuan and Hochschild, 2017; Molina-García et al., 2018). A survey of over 800 bacterial proteomes discovered >2,000 potential bacterial prions linked to diverse functional roles such as cell adaptability and invasion (Iglesias et al., 2015; Pallarès and Ventura, 2017). Bacterial prion-like proteins have a characteristic pattern of multi-phylum distribution coupled to sparse, intermittent conservation across their evolutionary range (Harrison, 2019). About 5% of compositionally-biased dark matter in the TrEMBL protein database (i.e., regions that cannot be assigned as either structured or intrinsically disordered) are predicted to be prion-like domains (Harrison, 2018).

Here, we examine the conservation of sequence and of prion-like composition for sets of prion-forming and prion-like proteins from the budding-yeast *S. cerevisiae*. We discover that prion-like composition in *S. cerevisiae* is strongly correlated with prion-like composition when weighted evolutionarily over dozens of fungal species, for both prion-formers and other prion-like proteins. However, sequence-wise prion-forming domains generally evolve more quickly than other prion-like domains.

## Methods

### Data

**Figure 1:**
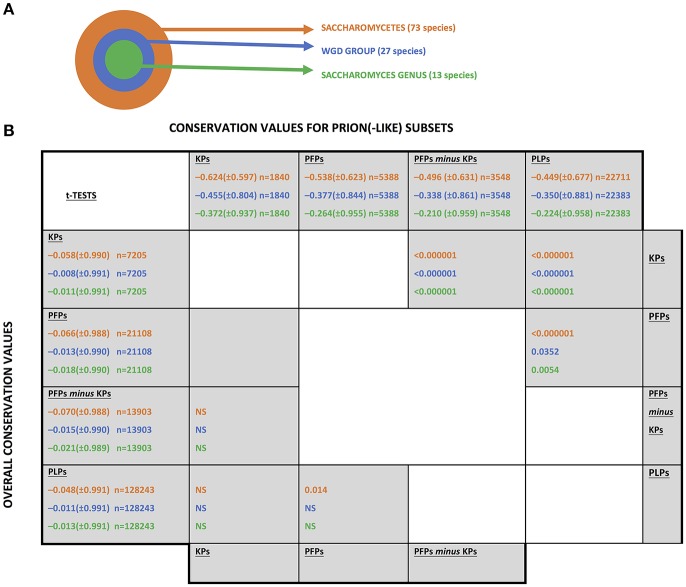
The fungal levels examined for conservation of prion(-like) proteins from *Saccharomyces cerevisiae*. **(A)** A schematic of the three taxonomic levels examined. “WGD” stands for “whole genome duplication.”**(B)** Conservation values of the different sets of prion-like or prion-forming domains. The abbreviations for the sets are as listed in section Methods. The mean conservation values for prion-forming or prion-like domains are listed along the top of the table, with the values for the whole sequences listed along the left-hand column. The standard deviations are in brackets. The number of coulmns in each sample is indicated (n). They are colour-coded as in part **(A)**. *t-*Tests were performed to compare the mean conservation values for each set, in the upper diagonal half for the prion(-like) domains and in the lower diagonal for the whole sequences of the same proteins. “NS” means “not significant”.

The UniProt (Boeckmann et al., 2003) set of reference fungal proteomes was downloaded from www.uniprot.org in June 2017, and collated into sets at three evolutionary levels relative to the budding yeast *S. cerevisiae*, as illustrated in Figure 1A.

### Sets of Prion-Forming and Prion-Like Proteins

Prion-forming proteins for *S. cerevisiae* were taken from the PrionHome database (Harbi et al., 2012; Harbi and Harrison, 2014). Two groups were analysed: (i) the set of *bona fide* known prions (the “KP” set), and (ii) a larger set made from these known prions plus other “prionogenic” amyloid-forming proteins (in total called the “PFP” set). The prion protein Mod5p, which underlies the [MOD+] prion state (Suzuki et al., 2012), is not included in the analysis, since it is not N/Q-rich. The PFP set includes the prionogenic proteins from the analysis of Alberti et al. (2009) that have been shown to form prions, through the SUP35C prion assay in conjunction with evidence for *in vivo* amyloid formation by the full-length proteins from other assays. The PFP protein set is as follows (UniProt IDs and standard gene names, the KP set is asterisked): P05453, Sup35^*^; P09547, Swi1^*^; P14922, Cyc8^*^; P23202, Ure2^*^; P25367, Rnq1^*^; P32432, Sfp1^*^; Q08972, New1^*^; P54785, Mot3^*^; Q02629, NUP100^*^; P32588, Pub1^*^; P40070, Lsm4; P14907, Nsp1; P18494, Gln3; P32770, Nrp1; P38180, YBL081W; P38216, YBR016W; P38429, Sap30; P38691, Ksp1; P40356, Pgd1; P53894, Cbk1; Q05166, Asm4; Q08925, Mrn1; Q12139, YPR022C; Q12221, Puf2; Q12224, Rlm1; Q12361, Gpr1; P40356, Med3; P12383, Pdr1; Q05672, Rbs1; P40956, Gts1; Q99383, Hrp1; Q06449, Pin3^*^.

A set of additional prion-like proteins was also generated. To do this, we applied the PLAAC prion prediction program (Toombs et al., 2012; Ross et al., 2013; Lancaster et al., 2014) to *S. cerevisiae*, and also applied it to all the complete fungal proteomes for further calculations of prion-like conservation as detailed below. PLAAC uses a Hidden Markov Model trained on the composition of known prion-forming domains, which all have a pronounced bias for N and/or Q residues. For PLAAC, we used PRDscore values ≥0.0 as prion propensity scores. Any other sequences analysed that yield negative values or “N/A” in the output from PLAAC are set equal to 0.0 for the purposes of this analysis. The boundaries for the predicted prion domain from PLAAC were used, if they have not been experimentally determined. The LLR score (which is the highest overall score achieved within a scanning window) has been used in previous studies to pick out the stretch of sequence in a protein that is most likely to form prions (Saupe, 2011; An and Harrison, 2016; Sideri et al., 2017; Harrison, 2019). We used PRDscore in preference to LLR score since it better reflects the overall degree of bias toward a prion-like composition (i.e., longer prion-like domains have higher PRDscore values).

### Multiple Sequence Alignment and Calculation of Conservation Scores

Orthologs in other fungal proteomes were detected for each *S. cerevisiae* protein examined using BLASTP and the bi-directional best hits method, with an expectation value threshold of 0.001 and default parameters otherwise (Altschul et al., 1997). Multiple sequence alignments of orthologs were performed using the default KMAD program (Lange et al., 2016), which is designed for optimal sequence alignment of intrinsically disordered proteins (Narasumani and Harrison, 2018), such as the N/Q-rich prion-forming proteins, which are the focus of this study. Sequence conservation was calculated using the program AL2CO with default parameters (Pei and Grishin, 2001). Mean conservation values were calculated as before for our analysis of folding-on-binding proteins (Narasumani and Harrison, 2015). Evolutionarily-weighted prion-propensity scores were derived according to the equation:

$$\frac{\sum_{i=1}^{n}\left(w_{i}\;*P_{i}\right)}{\sum_{i=1}^{n}w_{i}}$$

where ***P***_***i***_ is the prion propensity score for the **i**th ortholog, ***n***is the number of orthologs and ***w***_***i***_ is the evolutionary weighting for the **i**th ortholog sequence. This weighted score indicates the degree of conservation of the prion propensity score, but taking into account the differing divergences of the orthologs. Prion propensity scores = 0.0 are included in the summation. These evolutionary weightings were calculated in either of two ways. Firstly a “PC” (“%identity”) weighting was calculated ***w***_***i***_ = *(1 – %identity/100.0)*, where %identity is from the alignment of the *S. cerevisiae* protein and the ***i***th ortholog. Then a “BS” (“bitscore”) weighting was calculated ***w***_***i***_ = (**i***th bitscore / self-bitscore*) where the **i***th bitscore* is the bitscore from the alignment of the *S. cerevisiae* protein and the ***i***th ortholog, and the *self-bitscore* is the bitscore from aligning the *S. cerevisiae* protein to itself. The difference in the results from using these two weightings is minimal (as can be seen in Table 1). Generally, results for the PC evolutionary weighting are reported and discussed in the section Results and Discussion.

**Table 1:** Top 10 prion-like or prion-forming proteins by conservation across *Saccharomycetes* of prion-like composition, or of sequence^*^.

**UniProt accession**	**KP, *other* PFP or *other* PLP**	**Description**	**Score values**	
**Top 10 by Evolutionarily-weighted prion-like score (EWPS)**			**Score (PC) value**	**Score (BS) value**
Q02630	PLP	Nucleoporin NUP116/NSP116	159.7	160.5
Q02629	KP	Nucleoporin NUP100/NSP100	122.0	130.2
P35732	PLP	RNA polymerase II degradation factor 1 DEF1	121.4	119.3
Q03825	PLP	Transcription activator MSS11	109.4	108.2
P25367	KP	[PIN+] prion protein RNQ1	108.4	106.1
P09547	KP	SWI/SNF chromatin-remodeling complex subunit SWI1	90.8	90.7
P19659	PLP	Mediator of RNA polymerase II transcription subunit 15 GAL11	86.4	86.8
P05453	KP	Eukaryotic peptide chain release factor GTP-binding subunit SUP35	76.4	78.7
P48837	PLP	Nucleoporin NUP57	75.6	76.1
P32521	PLP	Actin cytoskeleton-regulatory complex protein PAN1	72.7	72.1
		**Top 10 by Sequence conservation score**	**Mean Value (±standard deviation)**	
P36041	PLP	EAP1 protein	0.295 (±0.868)	
P49687	PLP	Nucleoporin NUP145	0.290 (±0.701)	
Q02629	KP	Nucleoporin NUP100/NSP100	0.230 (±1.046)	
P80667	PLP	Peroxisomal membrane protein PAS20 (PEX13)	0.223 (±0.659)	
Q02630	PLP	Nucleoporin NUP116/NSP116	0.214 (±0.947)	
Q01560	PLP	Nucleolar protein 3 (NPL3)	0.177 (±0.754)	
P35732	PLP	RNA polymerase II degradation factor 1 (DEF1)	0.114 (±0.854)	
Q12118	PLP	Small glutamine-rich tetratricopeptide repeat-containing protein 2 (SGT2)	0.093 (±0.851)	
P47049	PLP	UBX domain-containing protein 6 (UBX6)	0.081 (±0.950)	
P53836	PLP	CCR4-NOT transcriptional complex subunit (CAF120)	0.058 (±0.939)	

* *For the EWPS, the two ways of calculating the score are tabulated (BS for bit-score, and PC for percent sequence identity). Proteins that are in the top 10s by both forms of conservation are underlined*.

Conservation of sequence and of prion-like status was examined at three evolutionary levels that are all centred on the *S. cerevisiae* species: (i) across the class *Saccharomycetes*; (ii) across the set of yeasts that descend from a common ancestor that underwent a whole-genome duplication (termed the “WGD group”); (iii) across the *Saccharomyces* genus (Figure 1A). This set of three levels was chosen because of the surge in formation of prion-like domains that has occurred since the last common ancestor of *Saccharomycetes* (An and Harrison, 2016). Also, the whole genome duplication may influence the conservation of prion status.

## Results and Discussion

### Overall Conservation Trends

Sequence conservation was analysed for known prion-forming regions and for other prion-like protein domains (PLPs) defined by the program PLAAC (Lancaster et al., 2014). The complete set of sequence conservation values is tabulated in Supplementary File 1. Prion-forming domains are considered either as a “known prion” domains (KPs) set, or a larger set of prion-forming domains (PFPs), of which the KPs are a subset. Mean conservation was compared for each of these three sets of domains (but using [PFPs-*minus*-KPs] when comparing to KPs) at three different evolutionary levels (Figure 1A). In general, regardless of evolutionary level, the KPs are significantly less conserved than the PFPs, which are significantly less conserved than the PLPs (Figure 1B, upper diagonal half). In comparison, there is virtually no significant difference detected for mean conservation values for the whole sequences of the proteins in which these domains reside (these mean conservation values include the components of the conservation that come from the prion-like/prion-forming domains; Figure 1B, lower diagonal half). Thus, these trends are due to mutation patterns in the prion-like/prion-forming areas themselves. So, for proteins with more evidence of prion-forming ability, the sequences have faster evolutionary rates. Such increased mutation rates may be linked to the acquisition of functional roles for prion-forming domains during the evolutionary epoch of *Saccharomycetes*. That is, out of the large “test set” of prion-like N/Q-rich domains that has formed during *Saccharomycetes* evolution (An and Harrison, 2016), we suggest that those that have become functional have mutated more quickly. These results also tally well with previous observations that intrinsically disordered regions often evolve more quickly than structured regions (Brown et al., 2002, 2011) (prion-forming regions tend to be highly intrinsically disordered; Harbi and Harrison, 2014; Harrison, 2017).

### Individual Conservation Behaviour of Prion-Forming Domains

**Figure 2:**
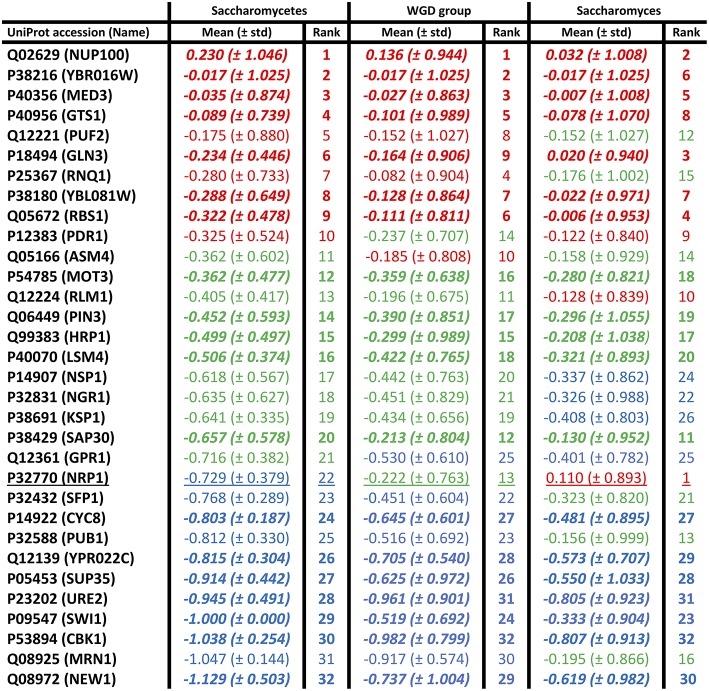
Individual mean conservation values for prion-forming domains. These are tabulated for each domain, with the top, middle, and bottom thirds colour-coded red, green, and blue, but with the ordering of the first column maintained across the figure. The numerical ranking is also listed for each domain at each level. The domains that are conserved in the same thirds at all levels are in bold italic. There is only one domain that moves between all three thirds of the list (underlined). The standard deviations are in brackets.

Mean conservation values were also calculated for each individual prion-forming domain, at the three evolutionary levels (Figure 2). The domains were grouped into thirds based on the ranking of their mean conservation values, as colour-coded in the figure. More than half of the prion-forming domains (59%) are maintained in the same third of the list across the three evolutionary levels (*Saccharomycetes*, the WGD group and the *Saccharomyces* genus). This indicates a substantial consistency in conservation across different evolutionary epochs in prion-forming domain evolution. Only one domain, in the protein NRP1, a domain from the Alberti et al. data set (Alberti et al., 2009), moves between the three thirds of the listings, to become the most conserved prion-like domain across the *Saccharomyces* genus (underlined in Figure 2). This is a putative RNA-binding protein that localizes to stress granules, which has not been studied extensively (Buchan et al., 2008). The five most conserved PFPs across the *Saccharomyces* genus sequence-wise also include: the GLFG-motif nucleoporin NUP100, a *bona fide* prion-former which is part of the nuclear pore complex (Halfmann et al., 2012); GLN3, a transcriptional activator of genes regulated by nitrogen catabolite repression; RBS1, a protein involved in assembly of the RNA polymerase III (Pol III) complex; MED3/PGD1, a subunit of the RNA polymerase II mediator complex. NUP100 is also a significant protein-interaction hub for other prion-like proteins (Harbi and Harrison, 2014). About a third of its interactors were found to be prion-like, with most of these being nucleoporins, including NUP116 and ASM4 (Harbi and Harrison, 2014). NUP116, which is the paralog of NUP100 arising from the whole-genome duplication that occurred during *Saccharomycetes* evolution, can be induced to form aggregate foci at low levels by over-expression of the prion domain of NUP100 (Halfmann et al., 2012). ASM4 is a member of the set of prion-forming proteins from the experiments by Alberti et al. (2009).

### Conservation of Prion-Like Composition

**Figure 3:**
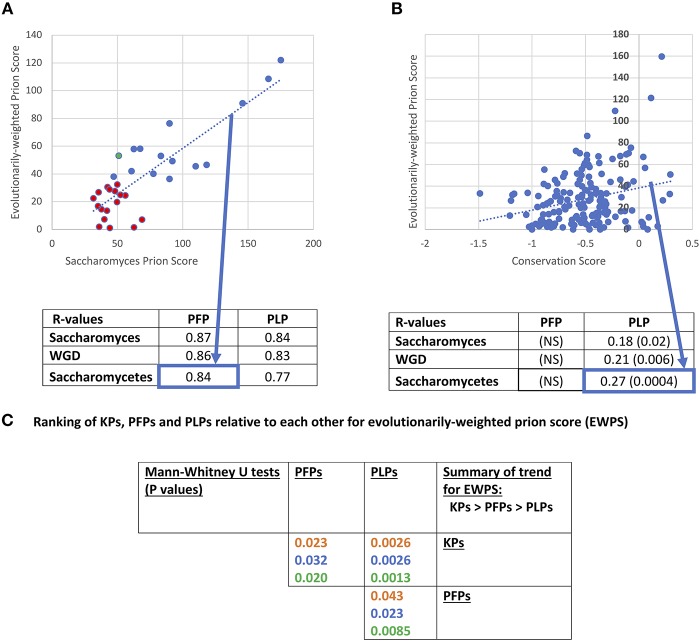
Evolutionarily-weighted prion score (EWPS). **(A)** Scatterplot of the EWPS vs. the *Saccharomyces cerevisiae* prion score (SCPS) for the prion-forming protein set at the *Saccharomycetes* level. Only one has an EWPS > SCPS (green point). Seventeen of the domains have EWPS < the lowest value of SCPS for a known prion domain (=35.6 for PIN3). Below the plot is a tabulation of *R*-values of these correlations for the PFP and PLP sets at the three evolutionary levels. These are all significant at *P* < 0.000001. The percent sequence identity method for calculating the EWPS has been used, but the difference in results obtained with the bitscore method in all cases is minimal. “NS” stands for “not significant.” **(B)** Scatterplot of the EWPS vs. the conservation score at the *Saccharomycetes* level for the prion-like protein set. Below the plot is a tabulation of R-values for the PFP and PLP sets at the three evolutionary levels. The significant *P*-values are in brackets. **(C)** Ranking of KPs, PFPs and PLPs relative to each other for evolutionarily-weighted prion score (EWPS). Tabulation of results of Mann-Whitney *U*-tests for comparison of the EWPS scores of the PFP, PLP and KP sets. Colour coding is as in Figure 1.

Prion-like status has been demonstrated experimentally to be largely composition-dependent, although special roles in prion propagation have been determined for specific parts of prion-determinant sequence or specific repeat patterns (Toombs et al., 2010; MacLea et al., 2015; Shattuck et al., 2017). Domains of prion-like composition also have roles in formation of stress granules and other biomolecular condensates (Jain et al., 2016; Franzmann et al., 2018). Methods (such as PLAAC) to annotate prion-like regions largely rely on detection of regions of proteins that are compositionally similar to known cases of prion-forming regions (Lancaster et al., 2014; Cascarina et al., 2017). We used PLAAC to analyse the conservation of prion-like composition in orthologs of prion-forming and prion-like proteins of *S. cerevisiae*. An evolutionarily-weighted prion score (EWPS) was calculated, which is a prion score made from terms for other orthologs/species weighted according to how far away from *S. cerevisiae* an ortholog/species is (as described in section Methods). Thus, orthologs from more similar species are given lower weightings, while those from more dissimilar species are given higher ones. We discover that this score is highly correlated with the *S. cerevisiae* prion score (SCPS) for both PFPs and PLPs (Figure 3A). The complete set of conservation values for prion-like composition are tabulated in Supplementary File 1.

Previously, it was shown that there was a major surge in formation of prion-like regions since the last common ancestor of *Saccharomycetes*, and that this seems to be linked to increased formation of runs of asparagine residues (An and Harrison, 2016). Thus, this is the most appropriate level at which to analyse patterns of conservation. At this level, R-values for correlation of EWPS and SCPS are = 0.84 for PFPs, and = 0.77 for PLPs, and are maintained at levels of *R* >0.84 for the WGD group and the *Saccharomyces* genus. However, for only one prion-former is the EWPS > SCPS (Figure 3A). This is probably because the PLAAC algorithm is trained on *S. cerevisiae* proteins, and does not necessarily imply that the prion-forming ability of the proteins is generally less in these other species. However, about half (15/32, 47%) have values that are above the lowest level observed for SCPS for any known prion.

Is this conservation of prion-like status correlated with sequence conservation? Consistent with the observations for decreased conservation for PFPs relative to PLPs, we find no correlation between sequence conservation score and EWPS for the prion-formers (Figure 3B). However, there are significant correlations for the prion-like proteins at all levels between EWPS and conservation score, with the most significant value at the *Saccharomycetes* level. This implies that the prion-like composition of the proteins is partly conserved in specific sequence motifs. In comparison, no significant correlations were observed for prion-forming or prion-like proteins between sequence conservation and the SCPS, implying that the correlations are derived from evolutionary information.

We also checked whether the EWPS has significantly different behaviour for the different sets of KP, PFP and PLP sets (Figure 3C). As would be expected since the PLAAC algorithm was trained on the PFP set, the un-weighted SCPSs are significantly higher for the prion-formers compared to the other prion-like proteins (*P*-values < 0.000001, Mann-Whitney *U*-test). However, they are also significantly higher for known prions compared to the rest of the PFP set (*P*-values < 0.000001, MWU-test). This may be a sort of knock-on effect, since most of the other PFP set members were found in a large-scale analysis to detect prion-forming domains, wherein selection for testing was guided by an earlier algorithm trained on a handful of *bona fide* known prions (Alberti et al., 2009). This indicates that further experimental procedures that do not cause such a bias or feedback will be necessary to discover more prion domains that might look quite different.

### Prion-Like Proteins That Are Highly Conserved in Sequence and Prion-Like Composition

We examined which proteins in the combined KP, PFP and PLP sets have the highest conservation in terms of sequence and of prion-like composition in *Saccharomycetes* (Table 1). Only three of the top ten sorted on the evolutionarily-weighted prion score are known prions, the rest being PLPs. Nucleoporins (components of the nuclear pore complex) figure prominently (3/10), with the two most conserved being the known prion-former NUP100, and the prion-like protein NUP116 (which was discussed above). Of course, the conservation of the prion-like domains of nucleoporins may be for other reasons, such as for facilitating interactions between pore proteins.

In the top 10 sorted by sequence conservation score, we also see nucleoporins figuring prominently (3/10). The most conserved prion-forming/prion-like sequence in this list is the EAP1 protein, that competes with eIF4G for binding to eIF4E and accelerates mRNA degradation by promotion of decapping. Three proteins are in the top ten by both methods (underlined in Table 1, NUP100, NUP116 and the RNA polymerase degradation factor DEF1), indicating that their prion-like composition is conserved in sequence motifs.

## Concluding Remarks

This analysis can be used to guide further experiments aimed at finding prion-forming proteins, both from *S. cerevisiae* and from other *Saccharomycetes* species. Of course, promising candidates from other species that are evolutionarily conserved can be studied in *S. cerevisiae* as a model system, using well-established techniques that have previously been applied to candidate prion-formers from other fungi (Edskes and Wickner, 2013), bacteria (Yuan and Hochschild, 2017), the sea hare *Aplysia californica* (Si et al., 2003), the plant *Arabidopsis thaliana* (Chakrabortee et al., 2016), and from humans (Kim et al., 2013). Well-conserved prion-formers may have functional roles linked to prion-like aggregation that have been maintained across many millions of years of evolution. For example, the yeast protein PUB1 has prion-like aggregation that is conserved in the prion-like domain of its human co-ortholog TIA1 (Li et al., 2014; An et al., 2016), which functions as a stress granule component (Gilks et al., 2004). Despite this, PUB1 is one of the prion-forming domains that evolves at a faster rate sequence-wise across *Saccharomycetes*, possibly indicating the influence of complex selection pressures on its mutation rates (Figure 2).

## Data Availability

All datasets generated for this study are included in the manuscript/Supplementary Files.

## Author Contributions

T-YS performed data analysis, prepared figures, and edited the paper. PH conceived the study, performed data analysis, prepared figures and tables, and wrote the paper.

### Conflict of Interest Statement

The authors declare that the research was conducted in the absence of any commercial or financial relationships that could be construed as a potential conflict of interest.

## Supplementary Material

Supplementary File 1 List of prion-forming and prion-like proteins with their conservation values as studied in this research article.
