# Supplementary material for: Conservation of Prion-Like Composition and Sequence in Prion-Formers and Prion-Like Proteins of Saccharomyces cerevisiae
Source: Front Mol Biosci. 2019 Jul 11;6:54. doi: 10.3389/fmolb.2019.00054 (PMC6639077; doi:10.3389/fmolb.2019.00054)
Supplement: Supplementary File 1 — List of prion-forming and prion-like proteins with their conservation values as studied in this research article. [file Table_1.DOCX]

#

# List of sequence conservation values and values for prion-like character

# ------------------------------------------------------------------------

#

# THE FORMAT IS AS FOLLOWS:

# $1 = PFP (prion-forming protein) OR PLP (prion-like protein)

# $2 = UniProt accession identifier

# $3 = MEAN conservation score across Saccharomycetes

# $4 = STANDARD DEVIATION for conservation score across Saccharomycetes

# $5 = MEAN conservation score across the WGD group

# $6 = STANDARD DEVIATION for conservation score across the WGD group

# $7 = MEAN conservation score across the Saccharomyces genus

# $8 = STANDARD DEVIATION for conservation score across the Saccharomyces genus

# $9 = prion propensity score (PRDscore) in S. cerevisiae

# $10 = evolutionarily-weighted prion score (EWPS) across Saccharomycetes using the PC method

# $11 = EWPS across Saccharomycetes using the BS method

# $12 = EWPS across the WGD group using the PC method

# $13 = EWPS across the WGD group using the BS method

# $14 = EWPS across the Saccharomyces genus using the PC method

# $15 = EWPS across the Saccharomyces genus using the BS method

PFP P05453 -0.9140 (¬±0.4423) -0.6252 (¬±0.9722) -0.5497 (¬±1.0315) 89.773 76.3828 78.661 112.869 113.088 108.919 108.806

PFP P09547 -1.0000 (¬±0.0000) -0.5191 (¬±0.6921) -0.3325 (¬±0.9038) 145.610 90.812 90.6919 125.672 126.533 123.121 122.657

PFP P12383 -0.3254 (¬±0.5235) -0.2374 (¬±0.7073) -0.1220 (¬±0.8396) 35.940 1.87228 1.72347 2.05585 1.76327 1.42791 0.543839

PFP P14907 -0.6181 (¬±0.5669) -0.4423 (¬±0.7633) -0.3366 (¬±0.8616) 49.609 19.7375 20.7624 30.6951 37.8721 21.9213 30.8611

PFP P14922 -0.8034 (¬±0.1867) -0.6447 (¬±0.6012) -0.4805 (¬±0.8950) 118.102 46.4091 42.5345 88.8177 92.2708 108.519 105.302

PFP P18494 -0.2339 (¬±0.4464) -0.1644 (¬±0.9062) 0.0196 (¬±0.9402) 31.739 22.4919 24.1242 37.6079 36.3831 43.9846 41.2037

PFP P23202 -0.9448 (¬±0.4907) -0.9610 (¬±0.9005) -0.8050 (¬±0.9239) 37.909 14.5316 12.2963 35.6827 29.3935 31.565 29.5896

PFP P25367 -0.2800 (¬±0.7334) -0.0824 (¬±0.9041) -0.1761 (¬±1.0021) 165.340 108.444 106.051 113.762 111.473 127.958 139.873

PFP P32432 -0.7676 (¬±0.2887) -0.4514 (¬±0.6044) -0.3226 (¬±0.8200) 46.992 37.9131 40.1372 26.5472 33.3404 37.2205 45.0076

PFP P32588 -0.8123 (¬±0.3300) -0.5161 (¬±0.6924) -0.1564 (¬±0.9988) 49.959 32.4766 31.3109 26.6049 22.4362 61.7668 64.6839

PFP P32770 -0.7289 (¬±0.3791) -0.2216 (¬±0.7634) 0.1097 (¬±0.8934) 89.663 36.2517 35.2891 42.9088 42.4646 28.5618 27.0434

PFP P32831 -0.6348 (¬±0.6270) -0.4512 (¬±0.8289) -0.3263 (¬±0.9882) 56.145 24.5917 22.7808 12.2486 12.4363 23.3229 21.6179

PFP P38180 -0.2878 (¬±0.6494) -0.1280 (¬±0.8642) -0.0218 (¬±0.9710) 77.361 40.17 42.2848 45.5071 44.4324 52.3697 53.956

PFP P38216 -0.0173 (¬±1.0247) -0.0173 (¬±1.0247) -0.0173 (¬±1.0247) 62.387 57.952 57.952 57.952 57.952 57.952 57.952

PFP P38429 -0.6568 (¬±0.5782) -0.2129 (¬±0.8039) -0.1295 (¬±0.9517) 44.156 1.08701 0.897892 6.24575 4.73743 11.8324 9.37123

PFP P38691 -0.6413 (¬±0.3348) -0.4336 (¬±0.6560) -0.4081 (¬±0.8026) 40.003 7.23472 6.75224 23.4612 21.8071 17.3877 18.5528

PFP P40070 -0.5059 (¬±0.3735) -0.4217 (¬±0.7648) -0.3208 (¬±0.8929) 52.356 24.8957 23.9993 52.3504 51.1562 45.6176 44.9629

PFP P40356 -0.0349 (¬±0.8743) -0.0271 (¬±0.8632) -0.0069 (¬±1.0076) 67.336 58.0888 58.4561 71.5913 74.6557 47.8272 50.4905

PFP P40956 -0.0898 (¬±0.7393) -0.1014 (¬±0.9892) -0.0781 (¬±1.0699) 51.092 53.1651 53.8045 48.5552 51.2113 43.6117 45.6157

PFP P53894 -1.0377 (¬±0.2543) -0.9819 (¬±0.7991) -0.8072 (¬±0.9125) 92.036 49.1942 47.9918 96.563 89.4201 94.8805 91.0618

PFP P54785 -0.3618 (¬±0.4774) -0.3591 (¬±0.6381) -0.2804 (¬±0.8207) 109.847 45.4989 43.5063 51.4706 47.8057 55.2188 45.7055

PFP Q02629 0.2296 (¬±1.0460) 0.1356 (¬±0.9440) 0.0321 (¬±1.0077) 174.989 121.989 130.227 215.488 214.895 225.338 222.955

PFP Q05166 -0.3616 (¬±0.6022) -0.1847 (¬±0.8080) -0.1575 (¬±0.9286) 42.001 13.4779 13.0251 26.0073 25.2332 38.0274 37.4961

PFP Q05672 -0.3220 (¬±0.4781) -0.1113 (¬±0.8113) -0.0059 (¬±0.9528) 42.430 30.5221 31.1827 44.2381 44.8498 33.9082 33.2893

PFP Q06449 -0.4523 (¬±0.5929) -0.3902 (¬±0.8510) -0.2964 (¬±1.0547) 35.610 26.7739 26.6532 18.5095 17.2514 27.0972 25.4711

PFP Q08925 -1.0465 (¬±0.1437) -0.9169 (¬±0.5738) -0.1949 (¬±0.8656) 35.082 16.9561 17.6648 9.75408 10.1681 10.3301 8.73593

PFP Q08972 -1.1288 (¬±0.5028) -0.7368 (¬±1.0041) -0.6189 (¬±0.9820) 43.879 28.8579 29.8965 39.219 42.9217 34.8459 40.2242

PFP Q12139 -0.8149 (¬±0.3041) -0.7046 (¬±0.5397) -0.5725 (¬±0.7072) 68.915 7.22295 7.26809 23.4772 22.5286 25.6833 25.5646

PFP Q12221 -0.1745 (¬±0.8796) -0.1523 (¬±1.0271) -0.1523 (¬±1.0271) 83.208 52.9799 52.5137 54.8989 54.4745 54.8989 54.4745

PFP Q12224 -0.4045 (¬±0.4165) -0.1955 (¬±0.6749) -0.1282 (¬±0.8392) 48.129 27.6646 27.6671 19.1629 19.8019 35.4067 33.3976

PFP Q12361 -0.7165 (¬±0.3815) -0.5301 (¬±0.6096) -0.4011 (¬±0.7819) 62.596 1.47538 1.02706 7.51642 5.50945 14.9497 10.9903

PFP Q99383 -0.4991 (¬±0.4974) -0.2993 (¬±0.9886) -0.2083 (¬±1.0381) 60.680 41.851 43.8495 49.1114 48.7705 49.157 47.8109

PLP P04050 -0.3534 (¬±0.6333) -0.2805 (¬±0.9636) 0.3921 (¬±0.7082) 26.422 29.5097 30.2562 21.8673 24.1348 34.4135 36.4162

PLP P04147 -0.3032 (¬±0.7505) 0.1721 (¬±0.8976) 0.0877 (¬±0.9470) 30.786 33.4083 33.5649 28.3911 27.8425 35.9408 38.5202

PLP P0C2I3 -1 (¬±0.0000) 0 (¬±0.0000) 0 (¬±0.0000) 21.457 0 0 -1 -1 -1 -1

PLP P11746 -0.7685 (¬±0.3521) -0.5729 (¬±0.6026) -0.5163 (¬±0.7060) 85.939 35.4003 37.8726 51.9936 56.9211 60.9545 64.9016

PLP P13185 -0.7958 (¬±0.2429) -0.3452 (¬±0.8919) -0.1994 (¬±1.1212) 11.675 16.0268 16.1116 16.4526 17.7028 28.9917 26.6911

PLP P13574 -0.5615 (¬±0.2350) -0.3963 (¬±0.6153) -0.2281 (¬±0.8946) 14.518 9.00033 8.45704 16.606 14.2467 27.5982 24.0134

PLP P14164 -0.711 (¬±0.3528) -0.6931 (¬±0.5721) -0.6395 (¬±0.7846) 14.083 1.90087 2.31605 2.25447 3.04152 5.91768 6.82269

PLP P14680 -0.8945 (¬±0.2017) -0.752 (¬±0.5336) -0.6311 (¬±0.7018) 37.274 22.4622 22.8175 28.8393 30.8357 33.5078 39.1414

PLP P16649 -0.9404 (¬±0.2706) -0.8894 (¬±0.6131) -0.5224 (¬±0.8806) 33.427 30.9373 29.2264 29.9078 25.0737 39.6348 34.157

PLP P18480 -0.3699 (¬±0.4995) -0.1486 (¬±0.8856) -0.233 (¬±0.9811) 124.015 61.4241 59.532 93.6916 91.8424 74.2355 73.1576

PLP P18899 -0.1261 (¬±1.0035) 0.0271 (¬±0.9740) 0.0041 (¬±0.9896) 89.531 39.4052 37.7283 69.46 67.3206 58.6898 60.8667

PLP P19659 -0.4846 (¬±0.5650) -0.2902 (¬±0.8317) -0.3526 (¬±0.9845) 62.946 86.3939 86.8304 69.0296 67.169 60.532 61.3986

PLP P20134 -0.3595 (¬±0.2725) -0.2999 (¬±0.8405) -0.2214 (¬±0.7935) 14.317 22.2019 21.6255 19.0552 19.9013 42.6151 43.2897

PLP P20424 -0.6454 (¬±0.6868) -0.2276 (¬±1.0581) -0.3995 (¬±1.0058) 37.072 33.7824 34.6233 29.4709 31.4546 24.2897 27.7703

PLP P20676 -0.1033 (¬±0.8255) -0.0771 (¬±0.9302) -0.0945 (¬±0.9878) 52.454 41.1457 41.0328 47.2481 47.0813 39.6348 39.6347

PLP P21339 -0.4664 (¬±0.4410) -0.6715 (¬±0.6164) -0.4591 (¬±0.9314) 27.749 48.6418 48.6017 83.0155 82.3613 55.0201 55.0998

PLP P21657 -0.8598 (¬±0.2437) -0.6894 (¬±0.5517) -0.3729 (¬±0.7651) 35.07 23.8718 22.8284 12.9626 11.1654 35.3237 34.8834

PLP P22082 -0.8831 (¬±0.2318) -0.9597 (¬±0.5781) -0.7648 (¬±0.7157) 53.064 55.4065 53.8776 46.736 43.4888 52.5239 44.1298

PLP P22148 -0.6885 (¬±0.3243) -0.7858 (¬±0.4741) -0.5884 (¬±0.7431) 19.014 18.6587 18.3553 12.1178 13.0355 9.1911 11.0578

PLP P22470 -0.4961 (¬±0.3612) -0.3169 (¬±0.6475) -0.2427 (¬±0.8736) 17.683 5.13891 5.04948 11.84 12.2708 22.2294 20.1465

PLP P22579 -0.7315 (¬±0.4907) -0.702 (¬±0.5961) -0.4345 (¬±0.8410) 28.244 28.7642 26.7276 30.5889 30.2667 44.3183 42.3303

PLP P23291 -0.8511 (¬±0.6837) -0.7735 (¬±1.0472) -0.6687 (¬±1.1484) 41.491 40.1676 43.2063 49.3433 54.3054 48.9125 56.1222

PLP P23292 -1.1661 (¬±0.2497) -0.9752 (¬±0.8220) -0.5331 (¬±1.0409) 22.247 33.0762 35.3038 49.7401 48.4653 36.321 39.4337

PLP P23293 -0.7473 (¬±0.1890) -0.8953 (¬±0.4799) -0.6904 (¬±0.7287) 24.498 9.80455 9.96921 32.2339 35.3302 7.90395 6.32083

PLP P24276 -0.8242 (¬±0.3859) -0.5271 (¬±0.7956) -0.2085 (¬±1.0259) 41.537 26.0746 26.3615 41.5819 41.5734 39.2746 39.0785

PLP P24814 -1 (¬±0.0000) -0.6246 (¬±0.5214) -0.2742 (¬±0.8203) 18.299 3.61173 3.60821 10.3885 10.455 12.202 11.1197

PLP P25302 -0.5207 (¬±0.3603) -0.309 (¬±0.9017) -0.3153 (¬±0.8502) 49.692 24.012 23.5512 10.3218 9.66395 18.9898 16.7056

PLP P25339 -0.511 (¬±0.3762) -0.1269 (¬±0.8322) -0.1974 (¬±0.9207) 42.775 20.3478 20.0575 28.0332 27.3294 33.2253 32.4449

PLP P25567 -0.5429 (¬±0.4616) -0.3436 (¬±0.7353) -0.1698 (¬±0.8546) 35.522 22.3191 22.0664 17.4043 16.9205 26.1276 24.7643

PLP P25644 -0.5382 (¬±0.5397) -0.6993 (¬±0.9391) -0.4912 (¬±1.0389) 29.698 50.0775 49.4761 70.9355 71.1009 49.8775 49.5852

PLP P25655 -0.8426 (¬±0.2950) -0.6916 (¬±0.6861) -0.384 (¬±0.9559) 19.805 24.7675 23.2752 18.4817 19.1686 27.1247 27.2732

PLP P29295 -1.1954 (¬±0.2205) -1.2251 (¬±0.6544) -0.7973 (¬±1.0001) 42.307 26.6764 24.7961 19.8527 20.5023 24.283 19.6116

PLP P30665 -1.0281 (¬±0.5532) -0.9367 (¬±1.1828) -0.8423 (¬±1.0187) 9.901 2.58486 2.69916 12.8636 12.6929 17.2624 17.2665

PLP P31384 -0.7054 (¬±0.4239) -0.7544 (¬±0.7194) -0.3189 (¬±0.9077) 46.338 48.5953 48.3549 42.8181 44.4004 46.9204 46.8234

PLP P32389 -0.39 (¬±0.3503) 0.094 (¬±0.9979) -0.1553 (¬±0.9423) 14.401 10.6365 10.3157 11.2665 10.4054 12.5617 11.2391

PLP P32505 -0.7473 (¬±0.3485) -0.8242 (¬±0.7527) -0.5881 (¬±0.7750) 61.33 16.8716 16.686 16.5784 15.7064 24.8924 22.2258

PLP P32521 -0.5882 (¬±0.5671) -0.263 (¬±0.8264) 0.0414 (¬±0.9572) 116.729 72.6962 72.0882 112.707 112.387 109.284 107.19

PLP P32790 -0.2456 (¬±0.7638) -0.1409 (¬±0.9394) -0.0929 (¬±1.0013) 58.225 65.2112 65.716 54.783 54.5757 64.7347 64.3522

PLP P32862 -0.1307 (¬±0.6186) -0.0373 (¬±0.8525) 0.0147 (¬±1.0091) 30.93 16.9607 17.592 34.3224 33.9069 21.1178 21.5242

PLP P32896 -0.9126 (¬±0.2729) -0.8896 (¬±0.5153) -0.9979 (¬±0.7056) 29.819 4.88893 4.82237 5.76283 5.80917 9.81089 9.58642

PLP P32900 -0.1673 (¬±0.6928) -0.1257 (¬±0.8689) -0.097 (¬±0.9723) 29.744 16.9422 16.9294 30.8305 31.2615 31.3717 30.7586

PLP P33329 -0.616 (¬±1.0400) -0.616 (¬±1.0400) -0.203 (¬±1.1273) 12.399 0.0752977 0.0733771 0.0752977 0.0733771 0.145871 0.17002

PLP P33417 -0.3777 (¬±0.6463) -0.3222 (¬±0.7664) -0.0674 (¬±0.9138) 137.618 67.4629 51.3943 126.768 125.842 138.393 135.889

PLP P33748 -0.3051 (¬±0.8717) 0.04 (¬±0.9668) 0.04 (¬±0.9668) 20.098 23.4238 27.8451 27.1802 27.2816 27.1802 27.2816

PLP P34216 -0.4304 (¬±0.5408) -0.1676 (¬±0.8950) -0.0893 (¬±0.8777) 21.066 6.21073 6.10284 16.4276 15.8637 13.3964 14.3764

PLP P34217 -0.3498 (¬±0.3637) -0.636 (¬±0.7550) -0.4363 (¬±0.8492) 33.416 51.2793 48.7636 52.638 51.6291 53.686 56.5818

PLP P34756 -0.7998 (¬±0.2427) -0.5703 (¬±0.6534) -0.5117 (¬±0.7799) 48.24 4.76817 4.06614 9.4021 6.56471 15.9899 12.6375

PLP P34758 -0.7634 (¬±0.3097) -0.3156 (¬±0.6840) -0.0444 (¬±0.8967) 96.919 42.6399 41.1279 125.715 127.111 82.5591 81.4812

PLP P34761 -0.5717 (¬±0.2814) -0.4934 (¬±0.7315) -0.3855 (¬±0.8093) 31.293 42.0568 39.0286 25.8366 25.9025 32.3715 33.1677

PLP P35190 -0.4658 (¬±0.3741) -0.3273 (¬±0.6936) -0.1683 (¬±0.8228) 24.093 26.7586 26.7287 26.6125 24.8923 37.1466 35.2854

PLP P35732 0.1142 (¬±0.8536) 0.1695 (¬±0.9735) 0.1848 (¬±0.9655) 205.018 121.362 119.284 149.543 143.855 166.3 157.925

PLP P36041 0.2947 (¬±0.8682) 0.3126 (¬±0.9149) 0.2958 (¬±0.9180) 25.655 50.7089 51.6724 50.1184 53.3818 50.5613 59.8403

PLP P36102 -0.8372 (¬±0.2954) -0.8453 (¬±0.5350) -0.6433 (¬±0.6352) 15.696 7.03321 6.60077 1.3881 0.968665 13.3521 12.1978

PLP P38042 -0.8208 (¬±0.2750) -0.7414 (¬±0.5507) -0.5924 (¬±0.7021) 17.873 2.00793 1.81253 8.24221 7.45482 1.25075 1.30171

PLP P38080 -0.7457 (¬±0.2321) -0.6733 (¬±0.5036) -0.553 (¬±0.7527) 61.398 16.1191 16.974 24.9729 26.1899 33.4874 33.1762

PLP P38236 -0.5436 (¬±0.3935) 0.0633 (¬±0.9154) 0.163 (¬±0.9710) 25.283 19.9104 19.4122 23.9745 23.2234 15.5254 15.687

PLP P38266 -0.0948 (¬±0.6294) -0.1485 (¬±0.8546) -0.1504 (¬±0.9263) 116.057 70.416 70.4817 92.3203 91.5269 137.888 138.576

PLP P38330 -0.9169 (¬±0.2958) -1.117 (¬±0.6939) -0.724 (¬±0.9334) 38.666 8.69856 8.05519 35.4424 34.0768 34.5386 37.9399

PLP P38631 -1.1836 (¬±0.8172) -1.1556 (¬±1.2434) -0.6939 (¬±1.1677) 23.892 31.8639 31.9499 40.6812 40.6198 44.724 44.4016

PLP P38699 -0.7849 (¬±0.3173) -1.0913 (¬±0.4570) -0.9435 (¬±0.7470) 21.916 5.36118 5.38261 0.639989 0.678931 9.16873 8.63466

PLP P38741 -0.51 (¬±0.2982) -0.1101 (¬±0.8174) -0.2037 (¬±0.8541) 30.497 10.7811 11.1579 19.4123 19.4676 10.501 11.0023

PLP P38753 -0.7634 (¬±0.2880) -0.6894 (¬±0.6720) -0.4221 (¬±0.8913) 13.715 31.7709 31.7815 34.8315 34.9927 34.9266 35.0454

PLP P38781 0.0356 (¬±1.0388) 0.0554 (¬±0.9500) 0.0554 (¬±0.9500) 19.242 5.57161 3.02306 14.2246 17.8628 14.2246 17.8628

PLP P38827 -0.6056 (¬±0.3411) -0.5985 (¬±0.6790) -0.552 (¬±0.7924) 26.62 4.0435 4.38209 16.2603 16.4646 5.83616 6.2158

PLP P38856 -0.5528 (¬±0.4161) -0.4352 (¬±0.7757) -0.2899 (¬±0.9240) 67.285 70.333 70.4346 72.2594 71.2555 60.7685 61.7146

PLP P38889 -0.9096 (¬±0.1691) -0.6652 (¬±0.6077) 0.1236 (¬±0.8533) 20.141 9.28419 6.84472 8.49347 9.10104 16.6257 13.6345

PLP P38968 -0.5904 (¬±0.5721) -0.3288 (¬±0.8756) 0.0036 (¬±0.9410) 15.922 11.2256 11.0545 10.4995 10.5247 13.9768 13.8992

PLP P38996 -0.1751 (¬±0.5917) -0.0476 (¬±0.8229) 0.1647 (¬±0.9457) 101.038 67.9091 68.8359 103.166 102.935 93.8311 93.8125

PLP P39001 -0.3259 (¬±0.4556) -0.2324 (¬±0.6971) 0.015 (¬±0.8580) 17.229 2.0358 2.15673 8.05049 9.41794 2.12866 0.418897

PLP P39008 -0.5339 (¬±0.4298) -0.5846 (¬±0.8207) -0.4843 (¬±1.0023) 35.223 55.6055 54.4779 62.1741 61.9575 50.3002 54.2487

PLP P39016 -0.703 (¬±0.2786) -0.6131 (¬±0.5890) -0.3596 (¬±0.9284) 49.033 34.3363 33.655 37.948 36.024 51.6676 51.2114

PLP P39517 -1.4857 (¬±0.3747) -1.4854 (¬±0.8846) -0.9901 (¬±1.1622) 31.611 33.2431 33.0633 43.8255 44.3604 32.2392 33.2331

PLP P39743 -1.0529 (¬±0.3201) -1.1212 (¬±0.6251) -0.7338 (¬±0.8930) 14.868 13.5793 14.2374 21.0329 22.5125 30.3764 30.5067

PLP P39927 -0.6968 (¬±0.3399) -0.6293 (¬±0.5529) -0.4348 (¬±0.7654) 18.045 20.8391 20.2769 33.3866 32.728 34.0653 33.4554

PLP P39935 -0.5323 (¬±0.4878) -0.2267 (¬±0.7459) 0.1976 (¬±0.8512) 35.196 31.8304 32.3265 24.8947 26.0801 9.13205 9.58448

PLP P39936 -0.4017 (¬±0.6384) -0.3478 (¬±0.9403) -0.1361 (¬±1.0135) 41.723 37.3664 38.2783 47.9288 49.0986 61.0492 61.0059

PLP P40002 -0.5536 (¬±0.3089) -0.3897 (¬±0.6573) -0.317 (¬±0.7140) 110.667 45.891 44.7633 74.0873 73.712 92.1368 93.3683

PLP P40073 -0.8417 (¬±0.4179) -0.6248 (¬±0.8937) -0.4827 (¬±0.9403) 12.776 4.05225 3.93319 6.04742 5.75393 11.4916 11.0727

PLP P40095 -0.2867 (¬±0.7802) -0.4389 (¬±0.8196) -0.2082 (¬±0.8493) 27.943 6.93657 5.21724 7.42415 5.50535 8.15521 7.68751

PLP P40159 -0.3162 (¬±0.7729) -0.2813 (¬±0.7713) -0.2956 (¬±0.8926) 36.983 25.8013 24.1469 35.0927 35.3447 31.8976 30.4721

PLP P40209 -0.3959 (¬±0.4536) -0.2321 (¬±0.7982) -0.0205 (¬±0.8356) 25.972 12.7505 12.7199 18.2072 19.2386 22.097 24.2422

PLP P40325 -0.632 (¬±0.3623) -0.6187 (¬±0.6478) -0.371 (¬±0.9205) 13.121 5.08685 4.84887 3.88573 3.50383 3.13958 3.54626

PLP P40357 -0.6692 (¬±0.4310) -0.6205 (¬±0.6435) -0.5444 (¬±0.7959) 36.153 27.4114 27.5639 23.0289 23.6484 22.2265 19.7421

PLP P40463 -0.4019 (¬±0.4562) -0.5352 (¬±0.6319) -0.2051 (¬±0.7735) 9.167 4.09922 4.51554 7.67013 8.38553 8.71841 9.80346

PLP P40467 -0.8804 (¬±0.1806) -0.878 (¬±0.5201) -0.6289 (¬±0.7151) 72.068 6.19885 5.74693 10.0972 9.50012 16.3683 16.9346

PLP P40482 -1.041 (¬±0.5388) -0.8854 (¬±0.8621) -0.5464 (¬±0.9242) 33.7 33.8257 34.3652 44.4321 44.3088 42.648 41.9415

PLP P40485 -0.8961 (¬±0.2309) -0.6207 (¬±0.8255) -0.3376 (¬±1.0139) 45.09 13.863 13.666 32.8996 31.1709 27.8866 23.5622

PLP P40489 -0.5026 (¬±0.3438) -0.4855 (¬±0.6410) -0.4448 (¬±0.8153) 9.741 5.92808 6.16673 16.9755 18.0329 22.4934 27.5058

PLP P40523 -0.4378 (¬±0.5153) -0.3953 (¬±0.5700) -0.3137 (¬±0.6499) 15.199 12.6294 11.2611 21.044 18.1191 19.2877 16.9847

PLP P40561 -0.4731 (¬±0.5923) -0.7726 (¬±0.4823) -0.1048 (¬±0.7625) 25.597 3.57563 3.77537 2.64932 1.85873 4.13124 3.22384

PLP P40568 -0.5445 (¬±0.4206) -0.1867 (¬±0.8408) 0.0871 (¬±0.9968) 21.268 8.5241 8.59465 19.2257 18.9896 18.4382 17.5934

PLP P40989 -0.3858 (¬±0.9693) 0 (¬±0.0000) 0 (¬±0.0000) 34.856 15.5418 18.7418 31.735 31.735 31.735 31.735

PLP P41696 -0.4039 (¬±0.3129) -0.2518 (¬±0.7877) -0.3051 (¬±0.8863) 52.512 33.4167 34.1003 27.5299 28.3652 23.1123 26.159

PLP P41813 -0.7359 (¬±0.3188) -0.4538 (¬±0.7189) -0.5925 (¬±0.9347) 18.418 26.2277 24.3982 13.0383 12.9962 18.3176 18.6512

PLP P43582 -0.4535 (¬±0.5864) -0.3409 (¬±0.8277) -0.1691 (¬±0.9456) 32.802 42.9458 42.3577 36.9328 36.4487 38.7943 39.1054

PLP P45976 -0.3636 (¬±0.4440) 0.0679 (¬±0.8751) 0.0925 (¬±0.9753) 31.868 26.664 27.158 29.3675 28.8093 29.0915 29.2205

PLP P47043 -0.3677 (¬±0.5142) -0.1675 (¬±0.8676) -0.1906 (¬±0.9005) 13.163 3.08454 3.04025 7.23574 7.5563 0.898759 0.196585

PLP P47049 0.0809 (¬±0.9502) 0.1836 (¬±0.7645) 0.1836 (¬±0.7645) 10.816 0.205133 0.363043 0.205133 0.363043 0.205133 0.363043

PLP P47135 -0.4488 (¬±0.4162) -0.3065 (¬±0.8985) -0.2369 (¬±0.7602) 30.009 32.293 32.6013 40.4771 40.449 31.2945 32.5676

PLP P48415 -0.3155 (¬±0.5231) -0.1705 (¬±0.7774) 0.235 (¬±0.9998) 18.385 20.2037 20.2825 13.7617 14.0006 17.2057 17.3787

PLP P48510 -0.3021 (¬±0.8548) -0.0707 (¬±0.9957) 0.0205 (¬±0.9804) 21.384 13.9544 14.3079 17.5858 19.8724 19.6426 24.1845

PLP P48562 -0.9477 (¬±0.2065) -0.8327 (¬±0.6621) -0.6473 (¬±0.8926) 50.02 20.6861 22.2868 46.6059 44.0514 41.2936 39.0253

PLP P48837 -0.0731 (¬±0.8896) -0.2815 (¬±0.9237) -0.06 (¬±1.0594) 60.7 75.5948 76.0818 83.8547 84.2057 106.637 108.669

PLP P49573 0.0403 (¬±0.7600) 0.1054 (¬±0.8976) 0.1063 (¬±1.0728) 12.171 2.62384 2.65566 8.88347 8.51248 0.258971 0.264073

PLP P49686 -0.0509 (¬±0.9246) -0.0259 (¬±0.9413) -0.022 (¬±0.9688) 55.644 44.3515 45.033 57.5984 58.6694 39.7821 39.6089

PLP P49687 0.2897 (¬±0.7005) 0.188 (¬±0.8835) 0.1388 (¬±0.9646) 35.418 32.7018 33.191 39.1234 39.0349 39.6012 43.1287

PLP P50109 -0.6255 (¬±0.3940) -0.2837 (¬±0.6946) -0.2734 (¬±0.9646) 48.13 8.93117 7.60104 20.6435 18.8971 15.4209 11.9671

PLP P50896 -0.483 (¬±0.5098) -0.4205 (¬±0.8995) -0.3392 (¬±1.0202) 63.053 35.9198 34.9138 57.0062 53.1606 62.7114 59.1838

PLP P52960 -0.3624 (¬±0.7573) -1.128 (¬±1.5040) -1.128 (¬±1.5040) 18.679 0.0144247 0.020075 0.318065 0.406756 0.318065 0.406756

PLP P53050 -0.3711 (¬±0.4324) -0.3316 (¬±0.6424) -0.1618 (¬±0.8765) 17.161 6.31079 5.10803 6.08026 4.34341 9.68747 7.35698

PLP P53165 -0.5665 (¬±0.5349) -0.1115 (¬±0.8442) -0.0532 (¬±0.9459) 49.321 29.5807 30.9044 43.4737 41.9276 34.822 29.7019

PLP P53185 -0.3767 (¬±0.5276) -0.4256 (¬±0.6544) -0.4045 (¬±0.7615) 16.087 15.477 13.9687 24.8031 21.9263 21.4552 14.7885

PLP P53281 -0.29 (¬±0.7472) -0.0502 (¬±1.0039) -0.1009 (¬±0.9838) 27.135 16.3586 17.2134 29.0355 29.1499 32.717 32.7714

PLP P53297 -0.4437 (¬±0.4975) -0.1044 (¬±0.8185) -0.0908 (¬±0.9656) 34.835 39.1397 39.0236 45.3334 45.9506 46.4205 46.973

PLP P53309 -0.1162 (¬±0.8690) -0.036 (¬±1.0115) -0.1631 (¬±1.0150) 49.287 69.2692 68.8547 48.856 48.9207 71.3299 73.1814

PLP P53316 -0.3935 (¬±0.6226) -0.2342 (¬±0.7342) 0.1711 (¬±0.8835) 14.907 15.2973 15.0783 17.3034 16.564 7.69891 7.96541

PLP P53438 -0.4854 (¬±0.4476) 0.0405 (¬±0.9535) 0.0893 (¬±0.9318) 70.095 68.7869 67.2708 75.122 83.91 89.991 91.5102

PLP P53550 -0.4226 (¬±0.4533) -0.4086 (¬±0.7243) -0.2923 (¬±0.8713) 18.937 13.6224 13.0315 24.6057 21.008 18.8965 13.908

PLP P53617 -0.5318 (¬±0.4013) -0.2195 (¬±0.9243) -0.2351 (¬±0.9238) 28.934 39.957 38.8387 36.6132 40.4447 38.1039 44.2721

PLP P53829 -1.2076 (¬±0.1814) -1.2574 (¬±0.6127) -0.8438 (¬±0.9211) 37.649 12.7155 11.4042 18.883 20.5853 21.1394 18.6607

PLP P53836 0.0576 (¬±0.9394) -0.1038 (¬±0.9128) 0.0434 (¬±0.9879) 55.299 56.9661 56.0805 94.9009 95.5254 61.1561 63.1671

PLP P53845 -0.453 (¬±0.7357) -0.417 (¬±1.0111) -0.1348 (¬±1.0428) 22.911 27.6061 27.5151 30.2124 30.41 28.7281 28.577

PLP P53919 -0.4613 (¬±0.3271) -0.2701 (¬±0.7786) -0.181 (¬±0.9751) 53.083 57.8103 58.0526 71.012 71.1199 69.7202 69.4715

PLP P80667 0.2231 (¬±0.6592) -0.3191 (¬±0.6871) -0.0593 (¬±0.8765) 24.918 26.7863 26.8497 31.1972 32.3063 34.2716 38.8237

PLP Q00539 -0.3681 (¬±0.7730) -0.4266 (¬±0.9066) -0.1766 (¬±1.0335) 13.949 20.6841 20.1147 18.1075 18.479 15.4929 15.6802

PLP Q01477 -0.5609 (¬±0.5139) -0.3007 (¬±0.8059) -0.2356 (¬±0.8573) 45.437 23.2727 23.5108 45.8122 46.1385 49.0998 51.6211

PLP Q01560 0.1771 (¬±0.7543) -0.0475 (¬±0.8026) -0.0118 (¬±1.0228) 14.282 3.07389 2.22732 11.5095 11.3318 7.05131 5.10463

PLP Q02100 -0.329 (¬±0.3718) -0.3228 (¬±0.6091) -0.4333 (¬±0.6517) 14.939 22.0779 20.5176 8.13493 8.10004 9.61598 9.86211

PLP Q02199 0.0403 (¬±0.9102) -0.1075 (¬±0.9628) 0.0683 (¬±0.9940) 62.276 66.9569 67.4996 43.5616 43.5777 46.3723 47.0059

PLP Q02630 0.2137 (¬±0.9471) 0.1316 (¬±0.9522) 0.1755 (¬±0.9956) 186.365 159.667 160.506 177.545 177.096 156.164 152.861

PLP Q02792 -0.8894 (¬±0.3100) -0.9643 (¬±0.7705) -0.6297 (¬±0.8176) 36.629 42.0068 44.3673 59.0586 58.7239 70.5376 71.212

PLP Q02796 -0.1631 (¬±0.7848) -0.1024 (¬±0.8523) -0.0164 (¬±0.9198) 27.443 29.8858 31.2158 35.6706 38.2837 20.6974 22.5005

PLP Q03063 0.0246 (¬±0.8232) 0.0161 (¬±1.0219) 0.0315 (¬±1.0217) 16.062 6.98097 6.5151 10.0989 9.59669 17.4192 17.0014

PLP Q03088 -0.5058 (¬±0.4312) -0.1366 (¬±0.8451) 0.0021 (¬±0.8820) 39.52 61.1334 60.259 82.8557 82.803 86.6514 87.0942

PLP Q03213 -0.4325 (¬±0.4098) -0.1027 (¬±0.6849) 0.1662 (¬±0.8903) 22.017 13.4118 13.0748 11.3127 9.49316 22.4663 27.331

PLP Q03466 -0.5339 (¬±0.3807) -0.4512 (¬±0.6271) -0.3095 (¬±0.8830) 30.09 29.8939 29.8509 31.0206 31.0648 21.9438 21.8617

PLP Q03656 -0.8571 (¬±0.2139) -0.797 (¬±0.5506) -0.6485 (¬±0.5826) 24.639 2.56339 2.47203 3.27291 3.89068 13.7046 13.9959

PLP Q03667 -0.1241 (¬±1.0228) -0.155 (¬±0.9902) -0.2503 (¬±0.9913) 13.332 7.625 7.5272 7.59162 6.70987 8.54515 7.59286

PLP Q03735 -0.3031 (¬±0.9349) -0.2608 (¬±0.9282) 0.1014 (¬±0.9752) 37.656 13.8047 13.8506 29.3955 28.3187 25.8186 25.7122

PLP Q03761 -0.5762 (¬±0.3614) -0.7191 (¬±0.5543) -0.7833 (¬±0.6639) 43.203 51.6898 49.9257 36.0212 33.2374 23.3192 20.4977

PLP Q03825 -0.2224 (¬±0.5852) -0.3079 (¬±0.7222) -0.2928 (¬±0.8463) 159.27 109.394 108.163 102.913 98.8915 97.1247 90.6324

PLP Q03957 -0.8422 (¬±0.2047) -0.7563 (¬±0.5143) -0.6419 (¬±0.5766) 14.496 4.45737 4.5089 12.9379 12.7191 15.9775 15.4495

PLP Q04195 -0.5221 (¬±0.2879) -0.4366 (¬±0.6573) -0.2007 (¬±0.7888) 34.557 13.9901 14.3458 3.99585 2.18757 7.26045 4.01653

PLP Q05785 -0.513 (¬±0.8465) 0.1084 (¬±0.9403) 0.0824 (¬±0.8401) 76.151 59.307 56.5562 62.8279 59.1836 74.1768 79.1908

PLP Q05854 -0.8441 (¬±0.2879) -0.6554 (¬±0.5498) -0.5611 (¬±0.7356) 47.949 14.233 13.378 13.8845 13.3495 21.3686 22.0442

PLP Q05958 -0.0639 (¬±0.8479) -0.2749 (¬±1.0353) -0.2749 (¬±1.0353) 33.939 17.7475 17.0845 29.228 28.1645 29.228 28.1645

PLP Q06251 -0.7828 (¬±0.3565) -0.6493 (¬±0.6757) -0.4207 (¬±0.9652) 33.684 30.6664 31.5739 26.4832 35.7061 30.0122 48.4995

PLP Q06315 -0.4868 (¬±0.3483) -0.2533 (¬±0.7343) -0.2739 (¬±0.7385) 33.959 64.4834 63.8395 46.8928 48.9901 44.2307 42.7293

PLP Q07684 -0.4719 (¬±0.5083) -0.4119 (¬±0.6270) -0.2708 (¬±0.8511) 30.969 59.7093 60.1843 58.4276 58.9518 54.3437 54.3104

PLP Q07807 -0.6526 (¬±0.3385) -0.7146 (¬±0.6078) -0.5236 (¬±0.7304) 29.647 22.1058 20.8118 49.2863 45.7549 38.765 32.9679

PLP Q07834 -0.7956 (¬±0.2371) -0.4629 (¬±0.6450) -0.1948 (¬±0.8550) 13.802 2.95858 2.99222 5.04157 5.40244 4.62476 5.4111

PLP Q08157 -0.2658 (¬±0.4893) -0.2756 (¬±0.8400) -0.1489 (¬±0.9559) 22.584 43.8149 43.6925 61.1494 62.2202 56.0988 58.6672

PLP Q08601 -0.7853 (¬±0.5850) -0.8428 (¬±0.7260) -0.7604 (¬±0.8342) 59.745 45.6822 45.7966 43.7098 46.0598 49.4805 54.2784

PLP Q08831 -0.327 (¬±0.4490) -0.318 (¬±0.7037) -0.1406 (¬±0.9269) 32.922 15.6641 14.5655 37.3876 38.7712 27.6834 28.31

PLP Q08887 -0.2196 (¬±0.9211) 0.0448 (¬±1.0966) -0.0098 (¬±1.0167) 24.5 11.7059 11.0854 19.9109 19.6239 22.1151 21.5061

PLP Q12034 -0.3572 (¬±0.6577) -0.1894 (¬±0.9683) -0.1894 (¬±0.9683) 36.817 33.5216 33.3912 29.081 27.7793 29.081 27.7793

PLP Q12057 -0.9637 (¬±0.1703) -0.3801 (¬±0.7889) -0.1191 (¬±0.9406) 22.912 5.11174 4.93089 17.0862 17.7468 14.3445 14.7346

PLP Q12072 -0.9329 (¬±0.2287) -0.6773 (¬±0.5516) -0.1989 (¬±0.8389) 20.718 3.05649 2.89844 1.96827 1.98866 0.647154 0.317929

PLP Q12118 0.0926 (¬±0.8514) -0.0557 (¬±0.8595) -0.0463 (¬±0.9998) 12.694 11.3354 11.6128 6.34482 7.02244 10.7464 12.6906

PLP Q12124 -0.3816 (¬±0.5866) -0.4917 (¬±0.5980) -0.2942 (¬±0.8022) 44.67 12.2731 9.09255 18.3375 16.7325 26.3633 24.2413

PLP Q12151 -0.5841 (¬±0.3250) -0.6747 (¬±0.5394) -0.3062 (¬±0.8556) 48.995 12.2315 11.8557 26.6502 24.353 35.7354 36.3505

PLP Q12269 -0.4611 (¬±0.8445) 0 (¬±0.0000) 0 (¬±0.0000) 21.457 0 0 -1 -1 -1 -1

PLP Q12329 -0.1 (¬±0.7281) -0.1536 (¬±0.9553) -0.084 (¬±1.0515) 14.962 1.56187 1.4129 2.42063 2.34767 1.12496 0.0928773

PLP Q12446 -0.5028 (¬±0.6299) -0.399 (¬±0.8707) -0.3186 (¬±0.9726) 42.048 36.4595 37.9743 54.3249 56.9608 53.9255 61.9274

PLP Q12489 -0.1728 (¬±0.9851) -0.0486 (¬±1.0480) -0.2753 (¬±0.9910) 43.795 45.905 45.1718 41.5602 42.4047 45.7323 46.8873

PLP Q12518 -0.6925 (¬±0.5354) -0.4212 (¬±0.9742) -0.3216 (¬±1.0530) 40.906 51.8267 51.7241 46.0755 44.7632 53.301 54.4409

PLP Q12523 -0.539 (¬±0.5363) -0.6347 (¬±0.6778) -0.5623 (¬±0.8782) 14.178 6.32584 6.61442 15.0178 14.6741 9.04575 8.51041
